# Supplementary material for: Expression of the CIC-DUX4 fusion oncoprotein mimics human CIC-rearranged sarcoma in genetically engineered mouse models
Source: bioRxiv. 2023 Sep 28:2023.09.26.559519. Preprint. [Version 1] doi: 10.1101/2023.09.26.559519 (PMC10557731; doi:10.1101/2023.09.26.559519)
Supplement: Supplement 1 [file NIHPP2023.09.26.559519v1-supplement-1.pdf]

bioRxiv preprint doi: https://doi.org/10.1101/2023.09.26.559519; this version posted September 28, 2023. The copyright holder for this preprint (which was not certified by peer review) is the author/funder, who has granted bioRxiv a license to display the preprint in perpetuity. It is made available under aCC-BY 4.0 International license.

Supplementary data 1

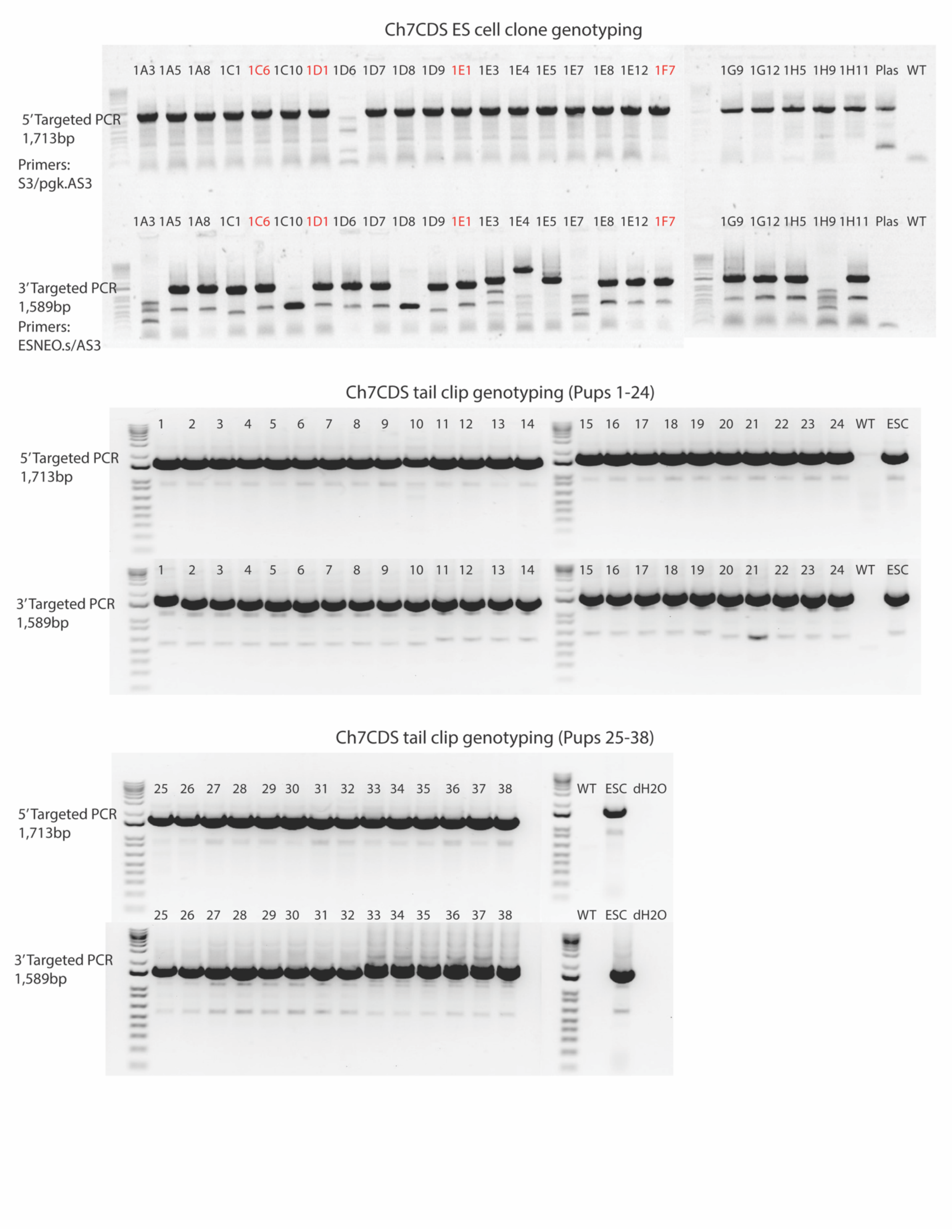

bioRxiv preprint doi: https://doi.org/10.1101/2023.09.26.559519; this version posted September 28, 2023. The copyright holder for this preprint (which was not certified by peer review) is the author/funder, who has granted bioRxiv a license to display the preprint in perpetuity. It is made available under aCC-BY 4.0 International license.

Supplementary data 2

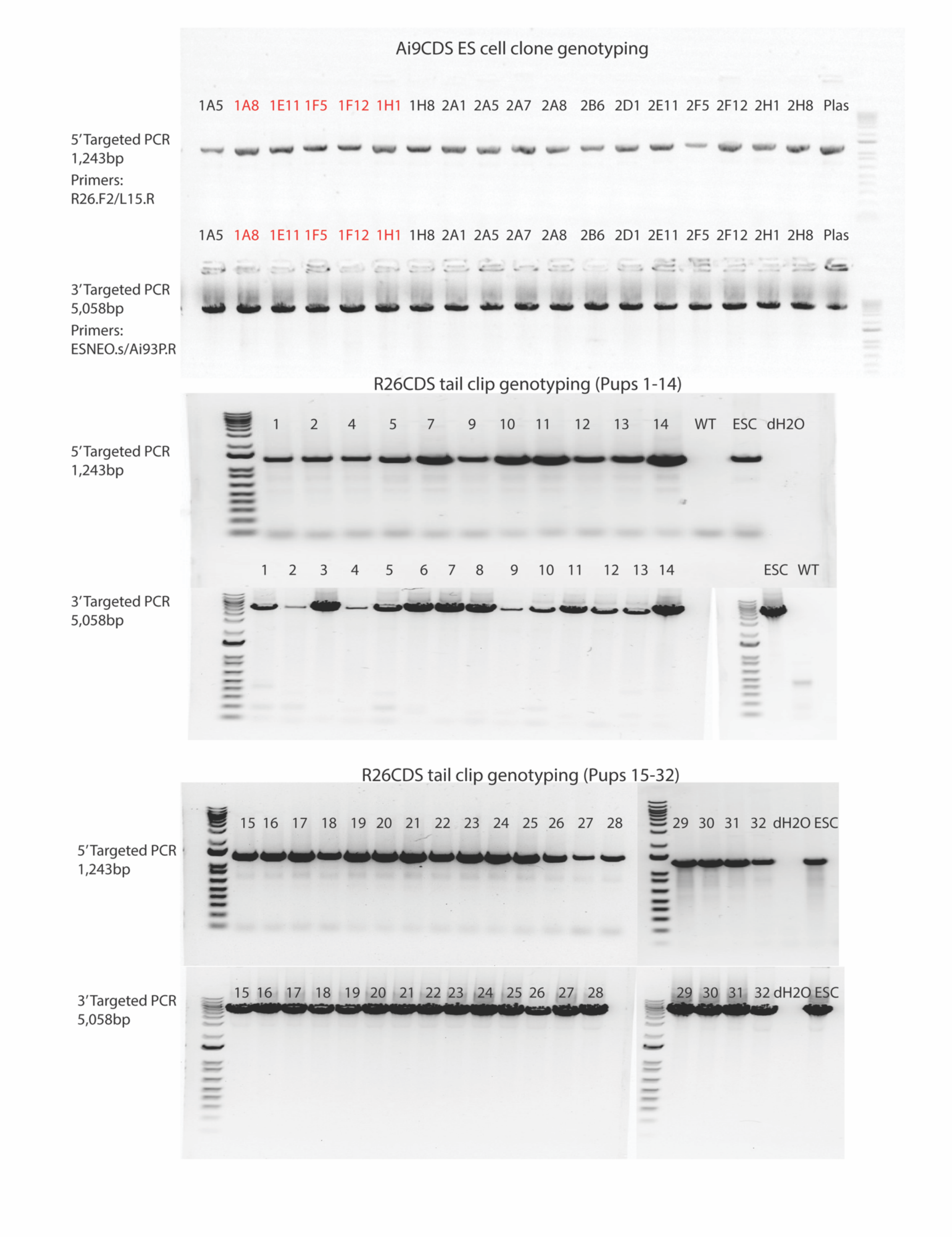

bioRxiv preprint doi: https://doi.org/10.1101/2023.09.26.559519; this version posted September 28, 2023. The copyright holder for this preprint (which was not certified by peer review) is the author/funder, who has granted bioRxiv a license to display the preprint in perpetuity. It is made available under aCC-BY 4.0 International license.

Supplementary data 3

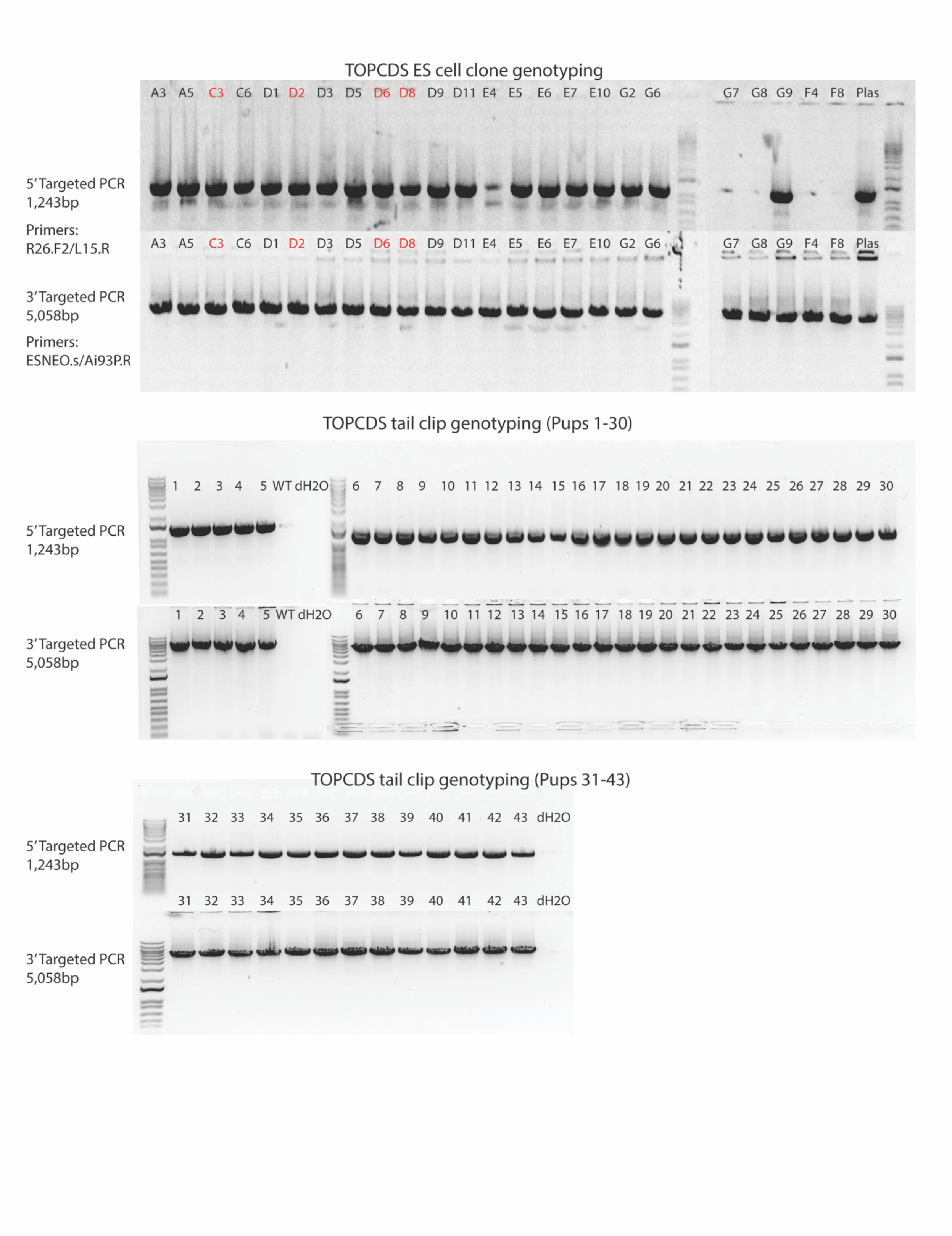

bioRxiv preprint doi: https://doi.org/10.1101/2023.09.26.559519; this version posted September 28, 2023. The copyright holder for this preprint (which was not certified by peer review) is the author/funder, who has granted bioRxiv a license to display the preprint in perpetuity. It is made available under aCC-BY 4.0 International license.

Supplementary data 4

**Ch7CDS**		
**S3**	5' integration site	5'-ACCCCCAAGAGTGCCAAGTGCGAG -3'
**Pgk.AS3**	5' integration site	5'-CTACCGGTGGATGTGGAATGTGTG-3'
**ESNEO.S**	3' integration site	5'-CTATCGCCTTCTTGACGAGTTCTTC-3'
**AS3**	3' integration site	5'-CCTGGTGGCAGTGCTGGCTTAGAC-3'
**1727F**	Recombination	5'-GAGACAGAGGATGTGCTCGG-3'
**5487R**	Recombination	5'-GAGCCCGGTATTCTTCCTCG-3'
**Ai9CDS/TOPCDS**		
**R26.F**	5' integration site	5'-CCTCAGAGAGCCTCGGCTAGGTAG-3'
**L15.R**	5' integration site	5'- TGGGCTATGAACTAATGACCCCGTA-3'
**ESNEO.S**	3' integration site	5'-CTATCGCCTTCTTGACGAGTTCTTC-3'
**AI93P.R**	3' integration site	5'-GACTTTAAGAGCCATGGCAATG-3'
**Cag.F1**	Recombination	5'-CGCAGCCATTGCCTTTTATGG-3'
**CicDuxR1**	Recombination	5'-TCAGAAGATGAGTCCCGTTCCTTG-3'
